# Molecular Epidemiology and Genotype Diversity of Severe Fever with Thrombocytopenia Syndrome Virus in Goats in South Korea

**DOI:** 10.3390/ijms27031264

**Published:** 2026-01-27

**Authors:** In-Ohk Ouh

**Affiliations:** Korea Disease Control and Prevention Agency, Osong-eup, Cheongju-si 28160, Chungcheongbuk-do, Republic of Korea; dvmoio@korea.kr

**Keywords:** tick-borne disease, goats, SFTSV, molecular epidemiology, genotype diversity

## Abstract

Severe fever with thrombocytopenia syndrome virus (SFTSV) is a tick-borne zoonotic pathogen of significant public health concern in South Korea, where human cases continue to occur at high levels; however, information on the molecular epidemiology and genotype diversity of SFTSV in goats—an increasingly important livestock species—remains limited. In this study, blood samples were collected from 750 clinically healthy goats during nationwide surveillance in 2024. Viral RNA was detected by RT-PCR targeting the S and M genomic segments. Epidemiological characteristics were analyzed according to season, region, farm size, breed, and sex. Positive samples were subjected to sequencing and phylogenetic analysis to determine SFTSV genotypes. SFTSV RNA was detected in 10 of 750 goats (1.3%), with significantly higher detection rates in autumn compared with summer, in southern regions compared with northern regions, and in female goats compared with males, while no significant association was observed with farm size or breed. Phylogenetic analysis showed that goat-derived SFTSV strains belonged to genotypes B2, D, and F; notably, genotypes D and F were identified in goats for the first time in South Korea. These findings indicate that goats are exposed to genetically diverse SFTSV strains circulating in tick populations and exhibit epidemiological patterns consistent with tick ecology and human SFTS incidence, supporting the role of goats as incidental or sentinel hosts. Continuous molecular surveillance of goats, integrated with vector monitoring programs, may enhance understanding of regional SFTSV transmission dynamics and facilitate early detection of emerging genotypes with public health implication.

## 1. Introduction

Severe fever with thrombocytopenia syndrome (SFTS) is a tick-borne zoonotic disease of significant public health concern in South Korea, characterized by severe clinical manifestations and a relatively high case fatality rate [1]. Since its first identification [2], the number of confirmed human SFTS cases has remained consistently elevated, with 172 cases reported in 2021, 193 in 2022, 198 in 2023, 170 in 2024, and a marked increase to 274 cases in 2025 [3,4].

As SFTS virus (SFTSV) is transmitted primarily by ticks, long-term national surveillance programs have been implemented by the Korea Diseases Control and Prevention Agency to monitor vector populations and assess viral circulation in the environment [5]. Since 2010, regional centers have been established to investigate the seasonal prevalence of disease vectors and vector-borne pathogens in relation to climate change [5,6,7]. These efforts have demonstrated widespread SFTSV infection in tick populations [5,8], as well as spillover into domestic animals and wildlife [8], highlighting the complex enzootic transmission cycle of the virus and the potential role of animals as indicators of viral circulation.

In parallel with these public health concerns, goat farming has expanded rapidly in South Korea in recent years due to changes in livestock consumption patterns, and multiple goat breeds now being raised, including native Korean black goats (*Capra hircus coreanae*), Boer goats (*Capra hircus*), and crossbred goats [9]. Goats are typically managed under outdoor or semi-grazing systems, which increases their exposure to tick-infested environments and places them at risk for tick-borne zoonotic diseases, including SFTSV and Q fever [9,10]. Previous studies have reported both molecular and serological evidence of SFTSV infection in goats; however, data on the epidemiological characteristics and genetic diversity of SFTSV circulating in goat populations remains limited.

Therefore, the present study aimed to investigate the prevalence, epidemiological characteristics, and genotype diversity of SFTSV in goats in South Korea, with particular attention to seasonal, regional, breed and host-related risk factors, as well as to the potential role of goats as sentinel hosts for circulating SFTSV genotypes.

## 2. Results

### 2.1. Identification of SFTSV in Goat Blood Samples

Specific Reverse transcription polymerase chain reaction (RT-PCR) amplification targeting the S and M genomic segments of SFTSV detected viral RNA in 10 out of 750 goat blood samples (1.3%; 95% confidence interval (CI): 0.5–2.2) (Table 1). Seasonal analysis showed that the prevalence of SFTSV was significantly higher in autumn (6/210; 2.9%; 95% CI: 0.6–5.1) compared with summer (4/195; 2.1%; 95% CI: 0.1–4.0) (*p* = 0.0293), while no positive samples were detected during spring or winter. Regionally, SFTSV was detected in the southern region (6/200; 3.0%; 95% CI: 0.6–5.4), followed by the central region (3/230; 1.3%; 95% CI: 0–2.8) and the northern region (1/320; 0.3%; 95% CI: 0–0.9). A statistically significant difference in SFTSV prevalence was observed between the southern and northern regions (*p* = 0.0341). With respect to farm size, SFTSV RNA was detected in small-scale farms (fewer than 50 heads; 8/420; 1.9%; 95% CI: 0.6–3.2) and medium-scale farms (50–199 heads; 2/280; 0.7%; 95% CI: 0–1.7). No significant association was observed between SFTSV detection and farm size (*p* = 0.2817). With respect to sex, the prevalence of SFTSV was significantly higher in female goats (8/320; 2.5%; 95% CI: 0.8–4.2) than in male goats (2/430; 0.5%; 95% CI: 0–1.1) (*p* = 0.0163). By breed, SFTSV RNA was detected in native Korean black goats (3/200; 1.5%; 95% CI: 0–3.2), Boer goats (2/230; 0.9%; 95% CI: 0–2.1), and crossbred goats (5/320; 1.6%; 95% CI: 0.2–2.9). No significant association was observed between SFTSV detection and goat breed (*p* = 0.7611).

### 2.2. Molecular Characterization and Phylogenetic Analysis

**Figure 1:**
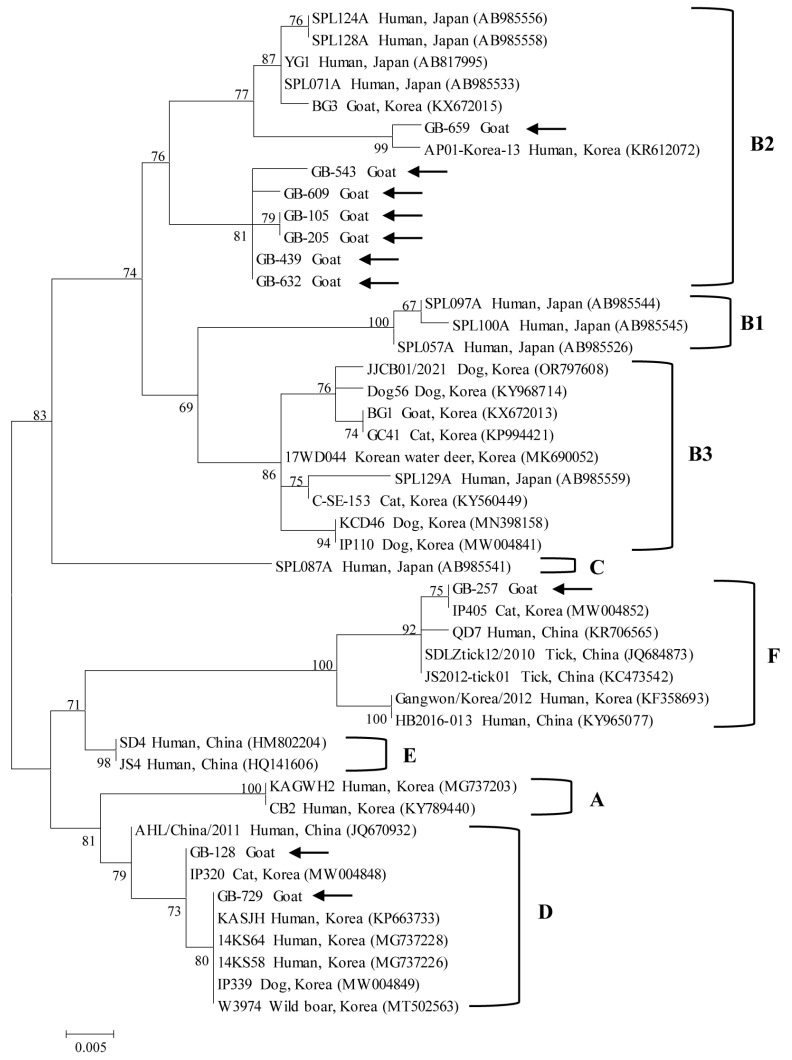
Maximum-likelihood phylogenetic tree based on partial nucleotide sequences of the S segment of severe fever with thrombocytopenia syndrome virus. Sequences obtained in this study are indicated by black arrows, while reference sequences retrieved from GenBank are labeled with their accession numbers. Genotypes (**A**–**F**) and subclades within genotype B (**B1**–**B3**) are shown on the right. Bootstrap values from 1000 replicates are displayed at the nodes. The scale bar represents the number of nucleotide substitutions per site.

**Figure 2:**
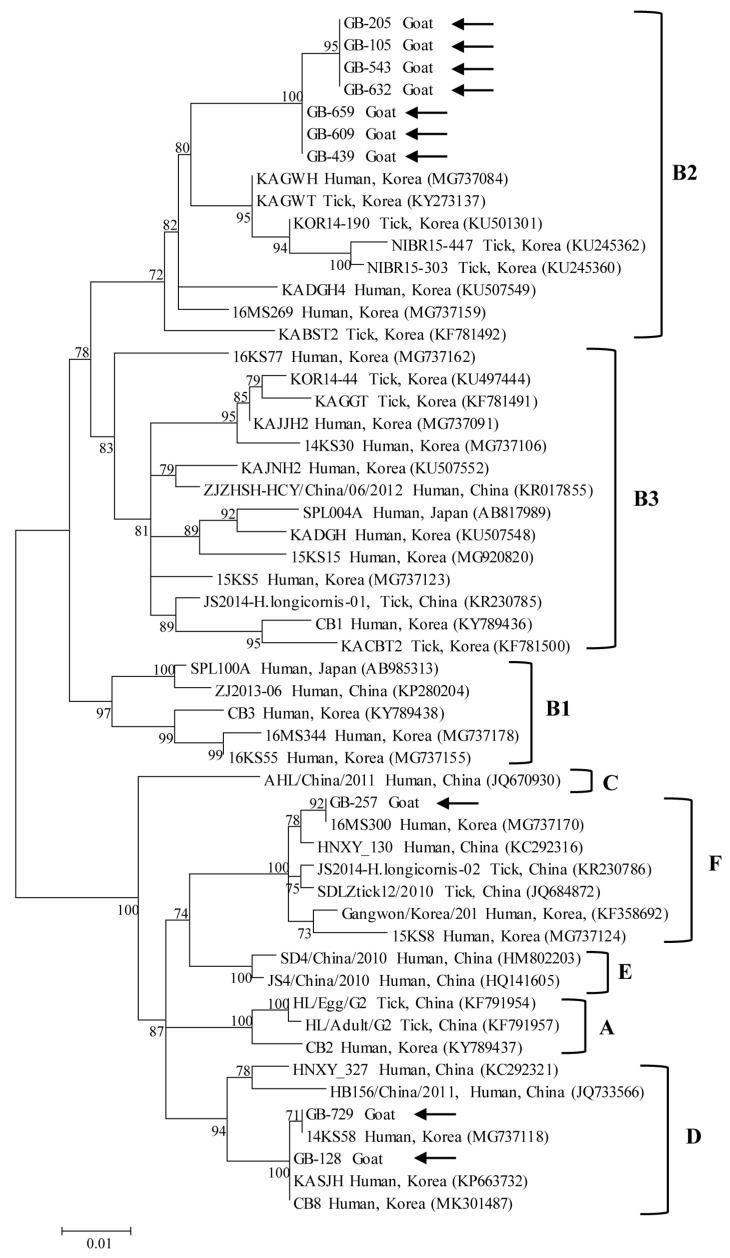
Maximum-likelihood phylogenetic tree based on partial nucleotide sequences of the M segment of severe fever with thrombocytopenia syndrome virus. Sequences obtained in this study are indicated by black arrows, while reference sequences retrieved from GenBank are labeled with their accession numbers. Genotypes (**A**–**F**) and subclades within genotype B (**B1**–**B3**) are shown on the right. Bootstrap values from 1000 replicates are displayed at the nodes. The scale bar represents the number of nucleotide substitutions per site.

Phylogenetic analysis based on partial nucleotide sequences of S segment (Figure 1) and M segment (Figure 2) demonstrated that the SFTSV strains identified in this study clustered with previously reported reference strains and were classified into three genotypes: B2, D, and F. Among the ten SFTSV-positive samples, genotype B2 was the most prevalent (70%, 7/10), followed by genotype D (20%, 2/10) and genotype F (10%, 1/10).

Pairwise genetic distance analysis was performed using the Kimura 2-parameter model. For the S segment (346 bp), intra-genotype genetic distances among genotype B2 strains ranged from 0.001 to 0.014, while genotype D strains showed distances of 0.002–0.003. In contrast, inter-genotype distances were markedly higher, ranging from 0.028 to 0.045 between genotypes B2 and D, and from 0.058 to 0.064 between genotypes B2 and F. Genotype F strains exhibited low intra-genotype genetic distances (0.000–0.006) but were clearly separated from genotypes B2 and D.

For the M segment (607 bp), intra-genotype genetic distances among genotype B2 strains ranged from 0.000 to 0.008, while genotype D strains showed a genetic distance of 0.003. Intra-genotype genetic distance for genotype F could not be estimated due to the presence of a single sequence. Inter-genotype distances ranged from 0.043 to 0.054 between genotypes B2 and F, from 0.054 between genotypes B2 and D, and from 0.008 to 0.013 between genotypes D and F.

Comparisons between study-derived sequences and GenBank reference strains revealed low genetic distances within the same genotype for both the S and M segments, supporting genotype assignments based on phylogenetic clustering. All genetic distance matrices were calculated using MEGA version 6.0 and are provided in Supplementary Tables S1 and S2.

The nucleotide sequences generated in this study were deposited in GenBank under accession numbers PX831869–PX831878 for the S segment and PX831879–PX831888 for the M segment.

## 3. Discussion

In South Korea, recent changes in livestock consumption patterns, including the ban on dog meat consumption, have resulted in a growing demand for alternative protein sources such as goat meat. Consequently, goat farming has expanded nationwide. As of December 2024, approximately 468,996 goats were raised across 11,474 farms, highlighting the increasing relevance of goats as potential hosts for zoonotic pathogens [11].

Previous surveillance studies in South Korea have demonstrated that widespread circulation of SFTSV among vectors [5,12], domestic animals [13,14,15], companion animals [16,17] and humans [18]. Viral RNA has been detected in mites [12], ticks [5], goats [14,15], dogs [17], cats [16,17], and cattle [13], while serological evidence indicates substantial exposure among livestock, particularly goats [14,15]. In goat, SFTSV infection has been documented by PCR (2.4%, 18/737) and ELISA (6.9%, 43/624) in nationwide surveys conducted in 2014–2015 [15], and by PCR (2%, 4/207) and ELISA (14.4%, 30/207) in regional studies performed in Chungbuk Province in 2017 [14], indicating ongoing exposure of goat populations to SFTSV.

In the present study, SFTSV RNA was detected in 1.3% of goat blood samples, and the detection rate varied significantly according to season, region, and sex, whereas no significant association was observed according to breed or farm size. These epidemiological patterns are consistent with the ecology of ticks, the primary vectors of SFTSV, and with previously reported temporal and geographical trends of SFTS in South Korea. Seasonal analysis revealed that SFTSV detection in goats was significantly higher in autumn than in summer, with no positive samples identified during spring or winter. This pattern corresponds closely with the seasonal dynamics of tick populations in South Korea [5]. Nationwide tick surveillance has shown that adult ticks activity peaks in early to mid-summer, while larval populations increase markedly from late summer to early autumn, with September representing a major peak for larvae. Notably, SFTS incidence in humans also reaches its maximum in autumn, particularly in October, suggesting that exposure to infected ticks during late summer and autumn plays a critical role in virus transmission. Goats grazing outdoors during this period are therefore more likely to encounter infected ticks, supporting the observed seasonal increase in SFTSV detection in autumn.

Regionally, SFTSV was detected most frequently in the southern region, followed by the central region, with the lowest prevalence observed in the northern region. This geographical pattern aligns with findings from previous nationwide tick surveillance studies [5], which have demonstrated higher tick densities and higher minimum infection rates of SFTSV in southern and southwestern regions of South Korea, including areas characterized by warmer climates, extensive grassland, and mountainous terrain. In particular, regions in the southern part of South Korea have been identified as hotspots for both tick abundance and SFTSV infection in ticks, which may increase the risk of virus exposure for grazing livestock such as goats.

With respect to farm size, SFTSV RNA was detected in small- and medium-scale farms, however no statistically significant association was observed. Small-scale farms often employ free-range or semi-grazing management systems, which may increase contact between goats and tick-infested environments such as grasslands, forest edges, and mountainous areas. However, the lack of a significant difference suggests that environmental exposure, rather than herd size alone, may be a more important determinant of SFTSV infection risk in goats.

A significantly higher prevalence of SFTSV was observed in female goats compared with males. Although the biological basis for sex-specific susceptibility to SFTSV in goats remains unclear, this finding may reflect differences in management practices rather than intrinsic host factors. Female goats are typically retained for breeding and milk production, resulting in prolonged outdoor exposure and cumulative contact with ticks over multiple seasons. In contrast, male goats are often slaughtered at younger ages, which may limit their opportunity for exposure to infected ticks.

With respect to breed, SFTSV RNA was detected in native Korean black goats, Boer goats, and crossbred goats, with no statistically significant differences among breeds. Although breed-specific differences in resistance to infectious and parasitic diseases have been reported in goats, particularly between indigenous and commercial breeds, such differences have been mainly documented for parasitic or bacterial infections rather than for tick-borne viral diseases [19]. To date, evidence supporting differential susceptibility to SFTSV among goat breeds remains limited. The absence of a significant association between breed and SFTSV detection in this study may suggest that exposure to infected ticks, rather than intrinsic breed-related host factors, plays a dominant role in SFTSV infection in goats. Given that all examined breeds in South Korea are commonly raised under outdoor or semi-grazing management systems, similar levels of environmental exposure to tick-infested habitats may account for the comparable detection rates observed among breeds, although further studies with larger sample sizes and immunogenetic approaches are warranted to clarify potential subtle breed-related effects.

Phylogenetic studies have shown that SFTSV strains circulating in South Korea can be classified into six major genotypes (A–F), with genotype B predominating in human infections and representing approximately 70% of reported cases. This genotype is further subdivided into B1, B2, and B3 subgenotypes, among which B2 is the most prevalent [18]. In contrast, other genotypes such as A, D, and F have been reported sporadically, while genotypes C and E have been detected only rarely [3].

Animal-derived SFTSV strains in South Korea have largely mirrored the genotype distribution observed in humans and ticks. Previous studies reported genotype B strains in mites [12], ticks [5], goats [14,15], dogs, and cats [17], while more recent investigations identified additional genotypes, including D and F, in companion animals [16] and grazing cattle [13]. These findings suggest that multiple SFTSV genotypes co-circulate among animal hosts in South Korea, reflecting ongoing viral diversification and genomic reassortment within the enzootic transmission cycle.

Notably, the present study identified SFTSV genotypes B2, D, and F in goats. To my knowledge, this represents the first report of genotypes D and F detected in goats in South Korea. This finding contrasts with earlier Korean goat studies, in which only genotype B strains were identified, and with reports from other countries. In China, goat-derived SFTSV strains have consistently clustered within the Chinese clade [20] and are primarily classified as genotype A or D according to the current genotype-based classification system, with no evidence of genotype F detection in goats. The broader genotype diversity observed in Korean goats, including the presence of genotype F, may reflect the high genetic diversity of SFTSV circulating in Korean tick populations. Indeed, nationwide studies have shown that ticks in South Korea harbor multiple subgenotypes of genotype B as well as other genotypes, with evidence of frequent reassortment, particularly in regions with high tick density and diverse wildlife hosts [5].

Taken together, these findings support the interpretation that goats act as incidental or sentinel hosts for SFTSV rather than as reservoirs of a single host-adapted viral lineage. The detection of multiple genotypes, including genotype F, in goats likely reflects repeated spillover events from infected ticks across diverse ecological settings. Given the expanding scale of goat farming in South Korea and the close contact between goats, ticks, and humans, ongoing molecular surveillance of goats may provide valuable insight into regional SFTSV circulation and serve as an early warning indicator for emerging genotypes with potential public health significance.

## 4. Materials and Methods

### 4.1. Ethical Approval

According to the Act on the Prevention of Contagious Animal Diseases (South Korea, amended 2025), national and local veterinary institutes conduct animal disease surveillance and control activities under annual infectious animal disease control programs. Blood samples used in this study were residual samples obtained from goats during routine Foot-and-Mouth Disease (FMD) seroprevalence surveillance conducted by licensed veterinarians at government-run local veterinary institutes. These blood samples were originally collected for official FMD monitoring purposes as part of regular health check-ups, and no additional handling or sampling of animals was performed specifically for this study. All samples were collected following verbal consent from farm owners, and blood collection procedures were carried out in accordance with the administrative guidelines of the Ministry of Agriculture, Food and Rural Affairs, South Korea. Since this study utilized only residual diagnostic samples collected through mandatory national disease surveillance and did not involve experimental animal procedures, institutional approval from an Institutional Animal Care and Use Committee was not required.

### 4.2. Sample Size Determination and Sample Collection

Sample size was determined by power analysis based on an expected disease prevalence of 10%, an absolute precision of 5%, and a confidence level of 95%, assuming a simple random sampling design [21]. Based on this sampling design, a minimum of 138 samples was required. A total of 750 blood samples were randomly collected from clinically healthy goats, including native Korean black goats, Boer goats, and crossbred goats, across multiple regions of South Korea during the national surveillance activities in 2024. Whole blood samples were collected in tubes containing ethylenediaminetetraacetic acid (EDTA). Information on farm size, geographic region, sex, and season at the time of sampling was recorded for subsequent analyses.

### 4.3. Molecular Detection of SFTSV

Total RNA was extracted from goat whole blood samples using the RNeasy Mini Kit (Qiagen, Melbourne, Australia) according to the manufacturer’s instructions. RT-PCR was performed using AccuPower RT-PCR Premix and AccuPower HotStart PCR Premix kits (Bioneer, Daejeon, Republic of Korea).

For molecular identification of SFTSV, the viral M segment (607 bp) was amplified using primers MF3 (5′-GATGAGATGGTCCATGCTGATTCT-3′) and MR2 (5′-CTCATGGGGTGGAATGTCCTCAC-3′) [22]. Reverse transcription was performed at 50 °C for 30 min, followed by initial denaturation at 95 °C for 15 min, and 35 cycles of 95 °C for 20 s, 58 °C for 40 s, and 72 °C for 30 s, with a final extension at 72 °C for 5 min. The S segment (346 bp) was amplified by nested RT-PCR using primers Np2F (5′-CATCATTGTCTTTGCCCTGA-3′) and Np2R (5′-AGAAGACAGAGTTCACAGCA-3′) in the first round, followed by primers Nn2F (5′-AAYAAGATCGTCAAGGCATCA-3′) and Nn2R (5′-TAGTCTTGGTGAAGGCATCTT-3′) in the second round, as previously described [8]. The first-round PCR consisted of reverse transcription at 50 °C for 30 min, initial denaturation at 95 °C for 15 min, and 40 cycles of 94 °C for 20 s, 52 °C for 40 s, and 72 °C for 30 s, with a final extension at 72 °C for 5 min. The second-round PCR was performed for 25 cycles of 94 °C for 20 s, 54 °C for 20 s, and 72 °C for 30 s, followed by a final extension at 72 °C for 5 min.

### 4.4. DNA Cloning

PCR amplicons were purified using the QIAquick Gel Extraction Kit (Qiagen) and ligated into pGEM-T Easy vectors (Promega, Madison, WI, USA). The recombinant plasmids were transformed into *Escherichia coli* DH5α competent cells (Thermo Fisher Scientific, Wilmington, DE, USA) and incubated overnight at 37 °C. Plasmid DNA was extracted using a plasmid miniprep kit (Qiagen) following the manufacturer’s protocol.

### 4.5. DNA Sequencing and Phylogenetic Analysis

The final alignment lengths used for phylogenetic reconstruction were 346 bp for the S segment and 607 bp for the M segment. Recombinant clones were selected and sequenced commercially by Macrogen (Seoul, Republic of Korea) using vector-specific primers. Nucleotide sequences obtained in this study were aligned with reference sequences retrieved from GenBank using CLUSTAL Omega version 1.2.1 (Bioweb, Ferndale, WA, USA). Sequence editing and trimming were performed using BioEdit version 7.2.5 (BioEdit, Manchester, UK) to remove ambiguous regions. Positions containing gaps or poorly aligned sites were excluded prior to phylogenetic analysis. Phylogenetic trees were constructed using MEGA version 6.0 [23] based on the maximum likelihood method with the Kimura two-parameter model [24]. Bootstrap analysis with 1000 replicates was conducted to assess the robustness of tree topology. Pairwise sequence comparisons were performed to evaluate nucleotide homology.

### 4.6. Statistical Analysis

Statistical analyses were conducted using GraphPad Prism version 5.04 (GraphPad Software Inc., La Jolla, CA, USA). Associations between categorical variables were evaluated using Pearson’s chi-square test. A *p*-value ≤ 0.05 was considered statistically significant. 95% confidence intervals were calculated for all estimates.

## 5. Conclusions

In conclusion, this study provides updated epidemiological and molecular evidence of SFTSV circulation in goat populations in South Korea. SFTSV RNA was detected in 1.3% of goat blood samples, with significant variations according to season, region, and sex. Higher detection rates in autumn, southern regions, and female goats were consistent with the ecological characteristics of ticks, the primary vectors of SFTSV, and with previously reported patterns of human SFTS incidence and tick activity in South Korea. Phylogenetic analysis revealed that goat-derived SFTSV strains belonged to genotypes B2, D, and F. Notably, this study represents the first report of genotypes D and F detected in goats in South Korea, thereby expanding the known host range of these genotypes. The detection of multiple genotypes in goats, including genotype F, suggests that goats are exposed to a genetically diverse pool of SFTSV circulating in tick populations and supports the interpretation that goats function as incidental or sentinel hosts rather than as reservoirs of a single host-adapted viral lineage. Given the recent expansion of goat farming in South Korea and the close ecological interface among goats, ticks, wildlife, and humans, goats may serve as valuable indicators of regional SFTSV circulation and emerging genotypes. Continuous molecular surveillance of goat populations, integrated with vector monitoring programs, will be essential to improve understanding of SFTSV ecology and to support early detection of viral genetic changes with potential public health implications.

## Figures and Tables

**Table 1:** Prevalence of SFTSV detected by PCR in goats in South Korea.

Group	Category	No. Tested	No. Positive (%)	95% CI ^1^	$\chi^{2}$ (df ^2^) (*p*-Value ^3^)
Seoson	Spring	185	0		9.000 (3) 0.0293
	Summer	195	4 (2.1)	0.1–4.0	
	Autumn	210	6 (2.9)	0.6–5.1	
	Winter	160	0		
Region	Northern	320	1 (0.3)	0–0.9	6.759 (2) 0.0341
	Central	230	3 (1.3)	0–2.8	
	Southern	200	6 (3.0)	0.6–5.4	
Farm size	Small	420	8 (1.9)	0.6–3.2	2.534 (2) 0.2817
	Middle	280	2 (0.7)	0–1.7	
	Large	50	0		
Sex	Female	320	8 (2.5)	0.8–4.2	5.767 (1) 0.0163
	Male	430	2 (0.5)	0–1.1	
Breed	native Korean black goat	200	3 (1.5)	0–3.2	0.546 (2) 0.7611
	Boer goat	230	2 (0.9)	0–2.1	
	crossbred goat	320	5 (1.6)	0.2–2.9	
Total		750	10 (1.3)	0.5–2.2	

^1^ CI: confidence interval. ^2^ df: degree of freedom. ^3^ *p*-values <0.05 were considered statistically significant.

## Data Availability

The original contributions presented in this study are included in the article/Supplementary Materials. Further inquiries can be directed to the corresponding author.

## Supplementary Materials

The following supporting information can be downloaded at: https://www.mdpi.com/article/10.3390/ijms27031264/s1.

## Abbreviations

The following abbreviations are used in this manuscript:

SFTS	Severe fever with thrombocytopenia syndrome
SFTSV	SFTS virus
FMD	Foot-and-Mouth Disease
RT-PCR	Reverse transcription polymerase chain reaction
CI	Confidence interval
