# Supplementary material for: Molecular Epidemiology and Genotype Diversity of Severe Fever with Thrombocytopenia Syndrome Virus in Goats in South Korea
Source: Int J Mol Sci. 2026 Jan 27;27(3):1264. doi: 10.3390/ijms27031264 (PMC12897668; doi:10.3390/ijms27031264)
Supplement: Supplementary file 1 [file ijms-27-01264-s001.zip › Table Caption.pdf]

**Table S1:** Pairwise genetic distances among SFTSV S segment sequences calculated using the Kimura 2-parameter model. Values represent the number of substitutions per site. Analyses were performed in MEGA version 6.0.

**Table S2:** Pairwise genetic distances among SFTSV M segment sequences calculated using the Kimura 2-parameter model. Values represent the number of substitutions per site. Analyses were performed in MEGA version 6.0.
